# Commentary: A Tablet-Based Assessment of Rhythmic Ability

**DOI:** 10.3389/fpsyg.2021.607676

**Published:** 2021-07-20

**Authors:** Agnès Zagala, Nicholas E. V. Foster, Simone Dalla Bella

**Affiliations:** ^1^International Laboratory for Brain, Music and Sound Research (BRAMS), University of Montreal, Montreal, QC, Canada; ^2^Department of Psychology, University of Montreal, Montreal, QC, Canada; ^3^Centre for Research on Brain, Language and Music (CRBLM), Montreal, QC, Canada; ^4^Department of Cognitive Psychology, University of Economics and Human Sciences in Warsaw, Warsaw, Poland

**Keywords:** music, rhythm, movement, auditory-motor synchronization, assessment mobile technologies, individual differences

## Introduction

Humans are well-equipped to move along with auditory rhythms via finger or foot tapping, body swaying or walking (Leman et al., 2013; Sowiński and Dalla Bella, 2013). Individual differences in auditory-motor synchronization abilities (AMS) are observed in the general population (Repp, 2010; Sowiński and Dalla Bella, 2013; Palmer et al., 2014), and exacerbated by disorders (e.g., language/speech disorders, Lundetræ and Thomson, 2018; Ladanyi et al., 2020; Parkinson, Yahalom et al., 2004; Puyjarinet et al., 2019). Describing these individual differences can shed light on the cognitive mechanisms underlying the rhythm system in healthy and patient populations (Dalla Bella, 2020; Damm et al., 2020; Ladanyi et al., 2020).

Finger tapping to test AMS (Repp, 2005; Repp and Su, 2013) is used in test batteries like the Battery for the Assessment of Auditory Sensorimotor and Timing Abilities (BAASTA, Dalla Bella et al., 2017), and the Harvard Beat Assessment Test (H-BAT, Fujii and Schlaug, 2013). Tapping performance is typically measured in the lab with tapping pads or dedicated sensors, which afford high temporal precision (≤ 1 ms), but make the method quite unsuitable to be used outside the lab.

## Using Mobile Devices for Testing Rhythmic Abilities

**Figure 1:**
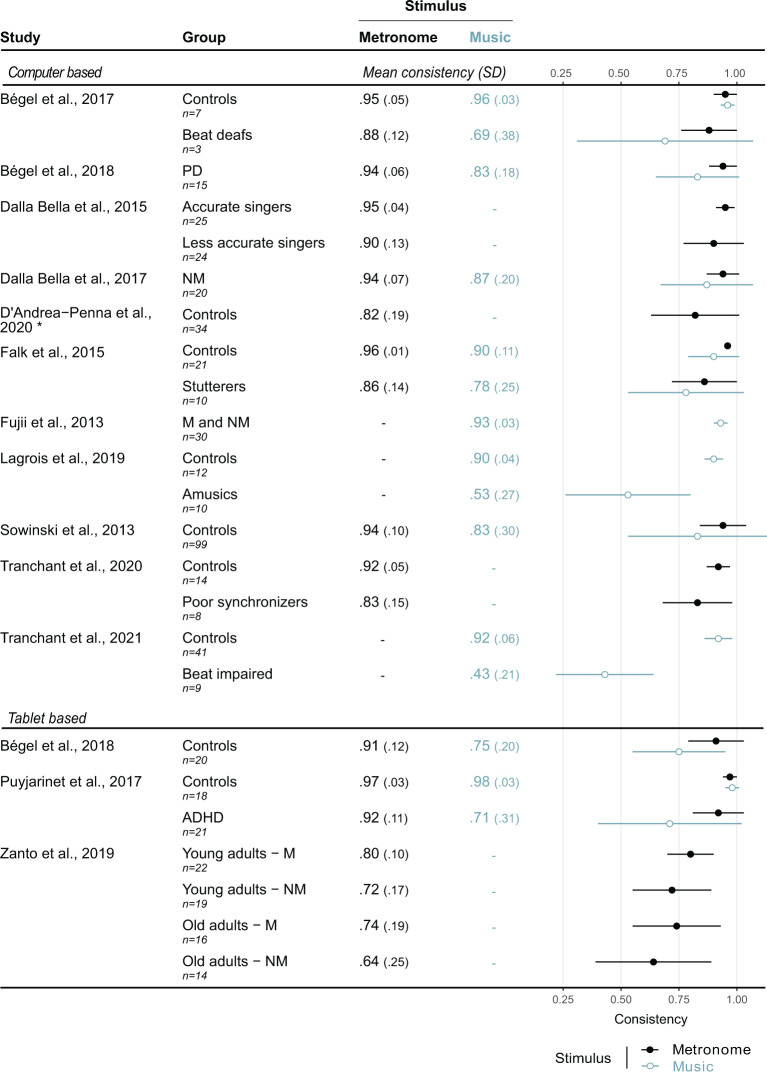
Comparison of synchronization consistency obtained during AMS tasks using a metronome or music. Synchronization consistency, a common measure of AMS, is a value from 0 to 1 (0 = lack of synchronization, high variability; 1 = perfect phase-locking between the taps and the beat, no variability). The results of AMS with a metronome are the mean of three different tempi that are comparable across studies (slow–around 450 ms, medium–around 600 ms, and fast–around 750 ms). The music stimuli were excerpts with an inter-beat interval around 600 ms. The dots on the plot represent the mean consistency, and the bars, the standard deviation. NM, non-musician; M, musician; ADHD, attention deficit disorder with or without hyperactivity; PD, Parkinson's disease; PS, poor synchronizers. *The task used in this study involved tapping to stimuli with 15% randomly omitted beats. Synchronization to these stimuli, less predictable than a standard metronome, may have yielded lower consistency values than in most of the other studies.

The portability of tablets and smartphones makes them an appealing solution for testing cognitive functions (Koo and Vizer, 2019), and rhythm abilities (tablet version of BAASTA; Puyjarinet et al., 2017; Bégel et al., 2018; Dauvergne et al., 2018). The study by Zanto et al. (2019) contributes to the demonstration that mobile technologies can serve purposefully for assessing AMS abilities. With their AMS task on tablet, Zanto et al. aimed at replicating outcomes of well-known AMS tasks, such as tapping to a metronome. The results are broadly consistent with previous studies, showing, for example, that musicians are more consistent than non-musicians in paced tapping (Franěk et al., 1991; Repp, 2010). Nevertheless, we notice that AMS performance (see Figure 1) is relatively low compared to other studies. This discrepancy may be linked to some of the limitations inherent in using touchscreen technology for tap detection.

## Limitations and Indications For Future Research

Timing inaccuracy in AMS tapping tasks on tablet can arise from: (1) a delay in the audio output, (2) the temporal uncertainty arising from the sampling rate of touch detection, and (3) the processing delay between the touch detection and the recording of a tap. Some of these limitations stem from the precision of the device touchscreen (sampling rate between 60 and 240 Hz) which is much lower as compared to lab measurements (1,000 Hz or more). Lower sampling rate imparts an unavoidable uncertainty about when the touch event occurred (Kousa, 2017). This variability (jitter) of error in individual touch events cannot be removed by subtracting an average delay. Because of this limitation, the participant's taps would appear more variable than their actual performance when measured in the lab. This hinders the capacity of the task to capture fine grained differences in AMS, and potentially to distinguish between good and poor synchronizers.

We compared the results from Zanto et al. (2019) with other in-lab studies and those using tablet devices (Figure 1), by taking synchronization consistency as a measure of variability in paced tapping (Fujii and Schlaug, 2013; Sowiński and Dalla Bella, 2013; Woodruff Carr et al., 2014). This measure corresponds to the vector length of the distribution of tap times within the inter-beat-interval, obtained with circular statistics. This measure shows high sensitivity to poor synchronization (e.g., Bégel et al., 2017; Lagrois and Peretz, 2019). Hence, synchronization consistency is a well-suited metric to assess the temporal precision of an AMS task, irrespective of constant latency in the task's technological implementation. It is apparent that non-musicians from Zanto et al.'s study generally obtained lower synchronization consistency (i.e., performed worse; mean consistency = 0.73; range = 0.64–0.80) than healthy adults from all the other studies (mean consistency = 0.93; range = 0.82–0.97) considered here. Their results are sometimes comparable to or show poorer synchronization than individuals with rhythm disorders (Bégel et al., 2017, 2018; Puyjarinet et al., 2017; Lagrois and Peretz, 2019). The observed generally lower synchronization consistency is likely to reflect low timing precision of touchscreen devices, a fact that may hinder quantitative comparison with validated norms and other laboratory-based studies of synchronization consistency. However, the performance reported by Zanto et al. is more comparable to values in the literature when considering other measures of tapping variability (e.g., standard deviation of asynchrony). In spite of these discrepancies, however, the precision afforded by Zanto et al.'s task is sufficient to distinguish musicians from non-musicians, and young from older adults, while providing high test-retest reliability. Thus, it may have general diagnostic value (e.g., for screening purposes). In addition, it is worth noting that Zanto's protocol also extends to visual and audio-visual synchronization, which is usually not tested by other batteries.

When synchronizing with audio stimuli, these issues with measurement precision on a tablet device can be solved by relying on an audio recording of the sound the taps produce when they reach the touchscreen. This solution, which is device-independent and capable of high temporal precision ( ≤ 1 ms) without requiring prior calibration, is already implemented in a tablet version of BAASTA (Dalla Bella and Andary, 2020). By recording the combined audio of stimulus and response, and resolving each during analysis, it bypasses possible sources of delay and jitter. This may explain why the tablet version of BAASTA successfully replicated the results previously obtained in the lab on a computer for AMS.

## Conclusion

Mobile devices such as tablets or smartphones are very promising methods for screening AMS abilities. Solutions based on audio recording can compensate the current limitations of mobile touchscreens, thus reducing measurement uncertainty and matching the precision of laboratory measurement.

## Author Contributions

AZ, NF, and SDB: wrote the manuscript. All authors contributed to the article and approved the submitted version.

## Conflict of Interest

The authors declare that the research was conducted in the absence of any commercial or financial relationships that could be construed as a potential conflict of interest.
